# The association between HIV stigma and HIV incidence in the context of universal testing and treatment: analysis of data from the HPTN 071 (PopART) trial in Zambia and South Africa

**DOI:** 10.1002/jia2.25931

**Published:** 2022-07-12

**Authors:** James R. Hargreaves, Triantafyllos Pliakas, Graeme Hoddinott, Tila Mainga, Constance Mubekapi‐Musadaidzwa, Deborah Donnell, Ethan Wilson, Estelle Piwowar‐Manning, Yaw Agyei, Nomtha F. Bell‐Mandla, Rory Dunbar, Ab Schaap, David Macleod, Sian Floyd, Peter Bock, Sarah Fidler, Janet Seeley, Anne Stangl, Virginia Bond, Helen Ayles, Richard J. Hayes, the HPTN 071 (PopART) study team

**Affiliations:** ^1^ Department of Public Health, Environments and Society, Faculty of Public Health and Policy London School of Hygiene and Tropical Medicine London UK; ^2^ Desmond Tutu TB Centre, Department of Paediatrics and Child Health, Faculty of Medicine and Health Sciences Stellenbosch University Cape Town South Africa; ^3^ Zambart, School of Public Health University of Zambia Lusaka Zambia; ^4^ Fred Hutchinson Cancer Research Center Seattle Washington USA; ^5^ Johns Hopkins University School of Medicine Baltimore Maryland USA; ^6^ Department of Infectious Disease Epidemiology, Faculty of Epidemiology and Population Health London School of Hygiene and Tropical Medicine London UK; ^7^ Department of Medicine, Imperial College NIHR BRC Imperial College London London UK; ^8^ Department of Global Health and Development, Faculty of Public Health and Policy London School of Hygiene and Tropical Medicine London UK; ^9^ International Center for Research on Women Washington DC USA; ^10^ Hera Solutions Baltimore Maryland USA; ^11^ Department of Clinical Research, Faculty of Infectious and Tropical Diseases London School of Hygiene and Tropical Medicine London UK

**Keywords:** HIV stigma, HIV incidence, cluster randomized trial, PLHIV, community members, health workers

## Abstract

**Introduction:**

To investigate the association between individual and community‐level measures of HIV stigma and HIV incidence within the 21 communities participating in the HPTN (071) PopART trial in Zambia and South Africa.

**Methods:**

Secondary analysis of data from a population‐based cohort followed‐up over 36 months between 2013 and 2018. The outcome was rate of incident HIV infection among individuals who were HIV negative at cohort entry. Individual‐level exposures, measured in a random sample of all participants, were: (1) perception of stigma in the community, (2) perception of stigma in health settings and (3) fear and judgement towards people living with HIV. Individual‐level analyses were conducted with adjusted, individual‐level Poisson regression. Community‐level HIV stigma exposures drew on data reported by people living with HIV, health workers and community members. We used linear regression to explore the association between HIV stigma and community‐level HIV incidence.

**Results:**

Among 8172 individuals who were HIV negative and answered individual‐level stigma questions at enrolment to the cohort, there was no evidence of a statistically significant association between any domain of HIV stigma and risk of incident HIV infection. Among the full cohort of 26,110 individuals among whom HIV incidence was measured, there was no evidence that community‐level HIV incidence was associated with any domain of HIV stigma.

**Conclusions:**

HIV stigma is often cited as a barrier to the effectiveness of HIV prevention programming. However, in the setting for the HPTN 071 “PopART trial,” measured stigma alone was not associated with the risk of HIV infection.

## 1 INTRODUCTION

HIV stigma is widely acknowledged as an important barrier to the success of HIV control efforts. Stigma acts as a barrier to HIV testing uptake, and, for those people living with HIV (PLHIV), to linkage to care, treatment initiation and adherence to antiretroviral therapy (ART) [1, 2, 3]. While stigma is also often cited as a barrier to the success of HIV prevention [4, 5], there is limited literature on this association.

A variety of plausible mechanisms might link HIV stigma with risk of acquiring HIV infection. At the individual level, perceiving that HIV stigma is present in communities or health settings, or anticipating that seeking HIV testing or HIV prevention services might lead to stigmatization, may put people at risk of HIV infection [6, 7]. HIV testing is an important gateway to HIV prevention service access. Alternatively, those who hold stigmatizing attitudes towards PLHIV may perceive themselves to be at low risk and take fewer precautions to avoid HIV risk. At the community level, if HIV stigma limits access to testing or treatment for PLHIV, this might limit the preventive impact of ART [8, 9]. Finally, at the structural level, HIV stigma is closely linked to a range of other prejudices, notably in relation to sexual practice. Homophobia, and other forms of prejudice and discrimination against those who may be vulnerable to HIV infection, for example, female sex workers, or adolescent girls and young women, might affect safe sex choices and access to preventive health services for these groups [10, 11].

In pre‐planned secondary analysis, we found that stigma has been gradually declining over time in Zambia and South Africa [12]. In this paper, we investigated the association between HIV stigma and risk of HIV infection among a large, representative population‐based sample in the 21 communities participating in the HPTN 071 (PopART) trial in Zambia and South Africa. We assessed (1) whether those who reported perceived stigma, or fear and judgement towards PLHIV, were at greater risk of new HIV infection and (2) whether those who lived in communities with higher levels of stigma were at greater risk of new HIV infection during the trial.

## 2 METHODS

### 2.1 Setting

The HPTN 071 (PopART) trial was a three‐arm cluster randomized trial conducted between 2013 and 2018 in 21 urban study communities (12 in Zambia and nine in Western Cape Province, South Africa) [13, 14]. We nested a mixed‐method study within the PopART trial to assess the effect of HIV stigma on HIV outcomes. We have reported the results of the association between HIV stigma and viral suppression among HIV‐positive participants [15]. In this paper, we present the results on HIV incidence among HIV‐negative participants. Details of the main and sub‐study designs have been described previously (Figure S1) [13, 16]. Briefly, study communities were arranged in seven triplets matched on geographical location and estimated HIV prevalence. Communities in each triplet were randomly allocated to three study arms. In the two treatment arms (A and B), a study‐employed cadre of community‐based health workers (HWs) known as Community HIV care Providers (CHiPs) delivered door‐to‐door HIV testing and referral services [17]. In Arm A, ART was offered to PLHIV regardless of CD4 count from the start of the trial; in Arms B and C, ART was offered according to national guidelines, which changed over the course of the trial and became regardless of CD4 count in 2016. HIV incidence was approximately 20% lower in Arms A and B combined than in the standard‐of‐care Arm C [14]. In all arms, health facility‐ and existing community‐based HWs received training on the study aims but did not receive specific anti‐stigma training. There was little evidence of a difference in stigma between arms at the end of the trial [12].

### 2.2 Outcome study population

The study population for this analysis was community members at risk of HIV infection who were recruited to a population‐based cohort (PC). In each community, one randomly selected adult aged 18–44 years was selected from a random sample of households. Enrolment mostly occurred between December 2013 and March 2015. Additional participants were enrolled in some study communities at 12 and 24 months, excluding households already sampled [14]. PC participants were surveyed at baseline (PC0) and at 12, 24 and 36 months (PC12/PC24/PC36). Laboratory‐based HIV testing was performed for all participants at all visits.

**Figure 1:**
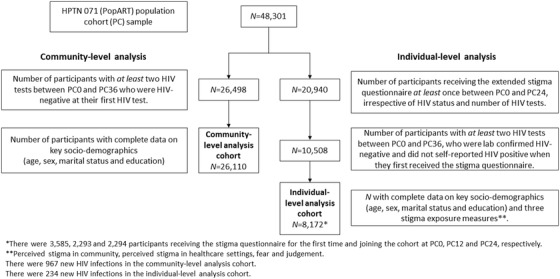
Flowchart with (a) the cohort‐level analysis cohort who had at least two HIV tests between PC0 and PC36 (*n* = 26,110) and (b) the individual‐level analysis cohort who received the stigma questionnaire at least once between PC0 and PC24 and had at least two HIV tests between PC0 and PC36 (*n* = 8172).

We analysed outcomes among two populations. First, for individual‐level analyses, questions on perceived stigma in community and health settings, and fear and judgement towards PLHIV, were asked of a 20% random sample of PC participants at each round. A new sample was drawn at each round. Participants entered the analysis cohort from the round at which they first answered questions about these three composite measures (domains) of stigma (Table S1), if at that round they were HIV negative and did not self‐report being HIV positive. To be included, participants also needed to have at least one further HIV test following the first test and complete data on socio‐demographic factors (age, sex, marital status and education) and HIV stigma measures in the round at which they joined. We refer to this group as the “individual‐level analysis cohort” (Figure 1). In total, 8172 individuals were included, joining the cohort at PC0 (*n* = 3585), PC12 (*n* = 2293) and PC24 (*n* = 2294).

Second, for community‐level analyses, we included all PC participants with at least two HIV tests who were HIV negative at their first HIV tests and had complete data on socio‐demographic factors (*n* = 26,110). We refer to this group as the “community‐level analysis cohort” (Figure 1). The individual‐level analysis cohort sample is a subset of the community‐level analysis cohort sample.

Blood samples were analysed in‐country using a single fourth‐generation assay (Architect HIV Ag/Ab Combo Assay, Abbott Diagnostics, Delkenheim, Germany). Further testing was performed at the HPTN Laboratory Center (HPTN LC, Johns Hopkins University, Baltimore, MD, USA). Samples that had reactive results in‐country were tested with a second fourth‐generation assay (GS HIV Combo Assay, Bio‐Rad Laboratories, Redmond, WA, USA). If seroconversion was confirmed, testing was performed to determine whether the participant had acute infection at the previous visit. HIV incidence was measured among participants who were HIV negative at enrolment to the cohort. HIV infection was assumed to occur at the midpoint between the last HIV‐negative sample and the first HIV‐positive sample. Imputation methods were used when the time of infection was unclear because of missed visits. The methods used have been previously described in the main trial [13]. For this paper, we used one of the imputed datasets (selected at random) to undertake the analysis on the basis that the imputation was about the timing of sero‐conversion, and not whether or not it occurred.

### 2.3 Measurement of stigma exposures

We used previously validated individual and community‐level composite stigma measures [18].

For individual‐level stigma exposures, we used three composite measures reflecting (1) perceived stigma in communities (five items) (2) perceived stigma in healthcare settings (two items) and (3) fear and judgement towards PLHIV (three items) [17]. Stigma items were pre‐coded using a 4‐item Likert scale (“Strongly agree” (3), “agree” (2), “disagree” (1) and “strongly disagree” (0)). For the primary analysis, all stigma items were collapsed into binary variables coded as “disagree” versus “agree.” This binary classification reflects whether participants agreed to any of the stigma items within each domain, compared to those who did not agree with any. In sensitivity analyses, we used the three composite stigma measures on a continuous scale, with values ranging from 0 to 3.

We developed community‐level measures of stigma using data from the community‐level analysis cohort above, and two further populations. At each round, we collected data from laboratory‐confirmed HIV‐positive PC participants who also self‐reported they were HIV positive. We developed four stigma measures reflecting community‐level stigma experienced by PLHIV in healthcare (three items) and community settings (five items), current internalized stigma (three items) and any stigma (combining the 11 items, Table S1). We used data collected at PC24 since this reflected the mid‐point of the trial. We also collected data from HWs (excluding CHiPs) self‐reporting not living with HIV in a separate cohort study, HWs which involved three rounds of data collection between July 2014 and February 2018; here, we used data from round 2 (R2) [15]. We developed three community‐level stigma measures reflecting perceptions of stigma by co‐workers in health facilities (four items), perceptions of stigma in the community (five items) and fear and judgement (five items, Table S1). Finally, we developed community‐level summaries of responses of participants in the community‐level analysis cohort to the individual‐level questions on stigma detailed in the previous section.

To develop community‐level summaries, for the data from PLHIV, each community was summarized with the % of PLHIV reporting each type of stigma. For the data from HWs and participants from the community‐level analysis cohort, we developed community‐level scores as the mean of the individual‐level scores. The scores thus had a theoretical range from 0 to 3 such that, for example, a mean score of 1 indicated that people in that community on average responded “Disagree” to stigma items and a mean score of 2 indicated people that on average responded “Agree.” Details of the item wording and other measurement details are reported elsewhere [18].

### 2.4 Statistical analysis

We first described the individual‐level analysis cohort comparing characteristics and stigma exposure measures between countries. We used chi square test to examine differences in the levels of stigma between those who were surveyed at baseline and those surveyed in later rounds (PC12 and PC24).

Participants’ characteristics from the individual‐ and community‐level analysis cohorts were similar (Table S2).

We then analysed the individual‐level association between the three domains of HIV stigma and HIV incidence between 0 and 36 months. We report the number of new HIV infections, total person‐years of observation, rate per 100 person‐years and calculated incidence rate ratios using Poisson regression. We developed an unadjusted and two adjusted models; the first adjusted for age group and sex, and the second adjusted additionally for marital status and education. All models were adjusted using community as a fixed term. We used interaction tests to explore whether the strength of these associations differed by trial arm and age. We estimated the predictive margins of HIV seroconversion for each interaction and plotted the probability of seroconversion with 95% confidence intervals. In sensitivity analysis, we run the same models described above using (1) the scores of the three composite stigma measures (instead of the binary measures) and (2) the 11 individual stigma statements using the binary classification.

We then analysed associations between community‐level stigma measures and HIV incidence between 0 and 36 months. We produced cluster‐level scatter plots to illustrate the strength of association between community‐level measures of stigma, expressed as scores (0–3) or percentages, and community‐level HIV incidence between 0 and 36 months. We used linear regression adjusting for trial arm, weighted by the sample size in each community, and report the *p*‐value for these associations.

### 2.5 Ethical considerations

Ethical approval for all study procedures was obtained from the institutional review boards of the London School of Hygiene and Tropical Medicine, Stellenbosch University and the University of Zambia. All participants provided written informed consent prior to enrolment.

## 3 RESULTS

The individual‐level analysis cohort included 8172 individuals of whom 70.6% were female, 44.5% were under 25 years of age, 71.2% had completed secondary education and 51.3% were unmarried (Table 1). Participants in Zambia were younger and more frequently female, married and with lower levels of educational attainment compared to participants in South Africa.

**Table 1:** Summary characteristics of the individual‐level analysis cohort (*n* = 8172), by country

	Zambia (*n* = 4766)		South Africa (*n* = 3406)		Total (*n* = 8172)	
	No.	%	No.	%	No.	%
Sex						
Male	1335	28.01	1064	31.24	2399	29.36
Female	3431	71.99	2342	68.76	5773	70.64
Age group (at PC0)						
16–24	2364	49.60	1271	37.32	3635	44.48
25–29	993	20.84	752	22.08	1745	21.35
30–34	665	13.95	555	16.29	1220	14.93
35–39	447	9.38	428	12.57	875	10.71
40+	297	6.23	400	11.74	697	8.53
Education (reported at first visit)						
Did not complete secondary	1400	29.37	426	12.51	1826	22.34
Completed secondary	3009	63.13	2813	82.59	5822	71.24
Further	357	7.49	167	4.90	524	6.41
Marital status (at enrolment)						
Married or living as married	2532	53.13	971	28.51	3503	42.87
Never married	1832	38.44	2361	69.32	4193	51.31
Divorced, separated or widowed	402	8.43	74	2.17	476	5.82
Any perceived stigma in the community^a^						
Agree, PC0 entry to cohort	1390/1917	72.51	841/1668	50.42	2231/3585	62.23
Agree, PC12 entry to cohort	890/1383	64.35	394/910	43.30	1284/2293	56.00
Agree, PC24 entry to cohort	910/1466	62.07	357/828	43.12	1267/2294	55.23
*p* value^b^				<0.01	<0.01	<0.01
Agree, all	3190/4766	66.93	1592/3406	46.74	4782/8172	58.52
Score (mean, SD)^c^	1.2	0.61	1.2	0.69	1.2	0.64
Any perceived stigma in healthcare settings^a^						
Agree, PC0 entry to cohort	543/1917	28.33	548/1668	32.85	1091/3585	30.43
Agree, PC12 entry to cohort	337/1383	24.37	226/910	24.84	563/2293	24.55
Agree, PC24 entry to cohort	301/1466	20.53	204/828	24.64	505/2294	22.01
*p* value^b^	<0.01	<0.01	<0.01			
Agree, all	1181/4766	24.78	978/3406	28.71	2159/8172	26.42
Score (mean, SD)^c^	0.9	0.66	1.1	0.69	1.0	0.67
Fear and judgement^a^						
Agree, PC0 entry to cohort	485/1917	25.30	304/1668	18.23	789/3585	22.01
Agree, PC12 entry to cohort	271/1383	19.60	188/910	20.66	459/2293	20.02
Agree, PC24 entry to cohort	296/1466	20.19	159/828	19.20	455/2294	19.83
*p* value^b^	<0.01	0.32	0.07			
Agree, all	1052/4766	22.07	651/3406	19.11	1703/8172	20.84
Score (mean, SD)^c^	0.8	0.58	0.9	0.58	0.9	0.58

Abbreviation: SD, standard deviation. PC0/PC12/PC24/PC36 population cohort at baseline, 12, 24 and 36 months.

^a^ Entry to cohort indicates the first time the stigma questionnaire was given to participants.

^b^ *p* value from chi square test looking at the differences in stigma measures over time by country and overall.

^c^ All scores have a theoretical range from 0 (all answers of all individuals “Strongly Disagree”) to 3 (all answers of all individuals “Strongly Agree”). A mean score of 1 indicates a person that, on average, responds “Disagree” to items within a score; a mean score of 2 indicates a person that on average responds “Agree.”

At cohort entry, 58.5% of participants from the individual‐level analysis cohort agreed or strongly agreed with at least one of five items reflecting perceived stigma in communities, 26.4% with at least one of two items reflecting perceived stigma in healthcare settings and 20.8% with at least one of three items reflecting fear and judgement towards PLHIV. Levels of perceived stigma and fear and judgement were higher in Zambia compared to South Africa. People recruited at later rounds were statistically significantly less likely to report any aspect of stigma, except fear and judgement in South Africa, than those recruited at earlier rounds (Table 1).

Participants were from communities with high HIV prevalence (range 3.0–35.6% at baseline) (Table 2). On average, 28.5% of PLHIV reported recent or current experience of at least one of 11 ways in which we measured stigma (range: 7.7–55.0%) (Table 2). Stigma in health settings was least commonly reported and varied least between communities. Community summaries of the responses of both community members and HWs not living with HIV on perceptions of stigma and fear and judgement towards PLHIV suggested that on average people “disagreed” with the statements provided, but with variation between individuals and communities.

**Table 2:** HIV prevalence and community‐level summaries of stigma, by country

	Zambia		South Africa		Total	
HIV prevalence^a^						
Baseline	21.0 (16.4–28.1)		21.2 (3.0–35.6)		21.1 (3.0–35.6)	
PC24	22.8 (16.5–30.9)		21.5 (3.6–36.1)		22.2 (3.6–36.1)	

Community‐level summary of stigma, using data collected from:	Community sample size (Arithmetic mean and range across communities)	Stigma prevalence*/score** (Geometric mean and range across communities)	Community sample size (Arithmetic mean and range across communities)	Stigma prevalence*/score** (Geometric mean and range across communities)	Community sample size (Arithmetic mean and range across communities)	Stigma prevalence*/score** (Geometric mean and range across communities)
Community members living with HIV						
Experienced stigma (any), %	222 (106–353)	33.2% (18.9–55.0)*	170 (12–298)	23.2% (7.7–50.0)*	199 (12–353)	28.5% (7.7–55.0)*
Internalized stigma, mean score	226 (120–356)	0.9 (0.5–1.3)**	171 (12–300)	0.8 (0.6–1.0)**	202 (12–356)	0.8 (0.5–1.3)**
Experienced stigma in the community, %	224 (112–356)	23.6% (13.5–45.9)*	171 (12–300)	15.9% (3.6–41.7)*	202 (12–356)	19.9% (3.6–45.9)*
Experienced stigma in healthcare settings, %	225 (107–354)	4.3% (1.8–14.0)*	172 (12–299)	5.9% (1.3–16.7)*	202 (12–354)	4.9% (1.3–16.7)*
Community members not living with HIV						
Perceived stigma in the community, mean score	205 (125–266)	1.2 (0.8–1.5)**	181 (146–211)	1.2 (0.8–1.5)**	195 (125–266)	1.2 (0.8–1.5)**
Perceived stigma in healthcare settings, mean score	205 (122–273)	0.9 (0.6–1.3)**	181 (140–212)	1.1 (0.7–1.3)**	195 (122–273)	1.0 (0.6–1.3)**
Fear and judgement, mean score	195 (122–250)	0.9 (0.5–1.2)**	175 (140–208)	0.9 (0.6–1.1)**	186 (122–250)	0.9 (0.5–1.2)**
Health workers, self‐reporting not living with HIV						
Perceived stigma among co‐workers in healthcare settings, mean score	65 (24–128)	0.8 (0.6–1.1)**	30 (11–44)	0.8 (0.6–0.9)**	50 (11–128)	0.8 (0.6–1.1)**
Perceived stigma in the community, mean score	68 (41–126)	1.2 (0.9–1.4)**	27 (10–39)	1.5 (1.3–1.7)**	50 (10–126)	1.3 (0.9–1.7)**
Fear and judgement, mean score	68 (43–128)	0.7 (0.5–0.9)**	27 (11–42)	0.7 (0.6–0.9)**	50 (11–128)	0.7 (0.5–0.9)**

Note: In community‐level analysis, measures of stigma were expressed as percentage/prevalence* or scores (0–3)** using the geometric mean. All scores have a theoretical range from 0 (all answers of all individuals “Strongly Disagree”) to 3 (all answers of all individuals “Strongly Agree”). A mean score of 1 indicates a person that, on average, responds “Disagree” to items within a score; a mean score of 2 indicates a person that on average responds “Agree.”

Abbreviations: HW, health workers; PC, population cohort; SR, self‐report. PC24, population cohort at 24 months.

^a^ Arithmetic mean and range in communities.

There were 234 new HIV infections observed during 16,401 person‐years (1.43 per 100 person‐years) in the individual‐level analysis cohort. There was no evidence of a statistically significant association between any of the three individual‐level stigma domains and HIV incidence (Table 3). We found no evidence that associations differed by trial arm or age (Figures S2 and S3). Results were similar when using the continuous stigma exposure measures (Table 3), and we found no evidence of an association when we used the individual stigma statements (Table S3).

**Table 3:** Association between HIV stigma and HIV incidence (PC0–PC36) in the individual‐level analysis cohort

Stigma measures	*N*/total person‐yr (rate per 100 py)	Unadjusted IRR	Adjusted IRR^a^	Adjusted IRR^b^
Cohort‐level analysis cohort (*n* = 26,110)	967/64,905 (1.49)			
Individual‐level analysis cohort (*n* = 8172)	234/16,401 (1.43)			
Any perceived stigma in the community				
Don't agree	100/6570 (1.52)	1	1	1
Agree	134/9832 (1.36)	0.90 (0.67–1.20)	0.91 (0.68–1.21)	0.92 (0.69–1.23)
Any perceived stigma in healthcare settings				
Don't agree	168/11,776 (1.43)	1	1	1
Agree	66/4625 (1.43)	1.05 (0.78–1.41)	1.07 (0.79–1.44)	1.06 (0.79–1.42)
Fear and judgement				
Don't agree	193/12,898 (1.50)	1	1	1
Agree	41/3504 (1.17)	0.82 (0.58–1.17)	0.83 (0.59–1.17)	0.83 (0.59–1.17)
Score		0.92 (0.73–1.17)	0.92 (0.73–1.17)	0.92 (0.73–1.17)

Abbreviation: IRR, incidence rate ratio; PC, population cohort.

Note: All models were developed within a Poisson regression framework adjusted using community as a fixed term. Each circle represents one community. Size of the circles is proportional to the number of participants in each community. Dashed lines reflect linear regression slopes from cluster‐level analyses of the associations and weighted by the size of the community in each cluster.

^a^Adjusted for sex and age group.

^b^Adjusted for sex, age group, marital status and education.

**Figure 2:**
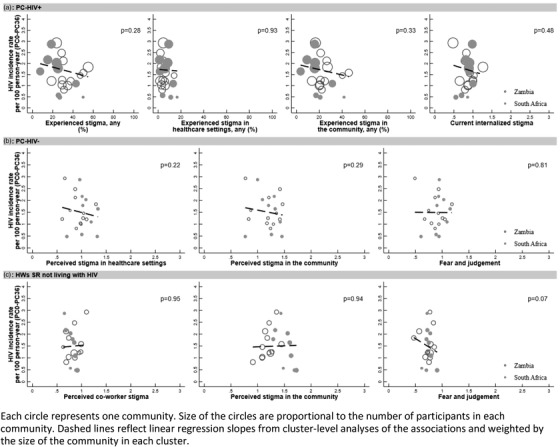
The association between levels of HIV incidence between PC0 and PC36 and (a) internalized and experienced stigma reported by people living with HIV, (b) beliefs and perceptions of community members not living with HIV and (c) beliefs and perceptions of health workers self‐reporting not living with HIV at PC24 and R2 in 21 communities in South Africa and Zambia.

In the community‐level analysis cohort of 26,110 individuals, a total of 967 new HIV infections were observed during 64,905 person‐years of follow up (1.49 per 100 person‐years). There was no evidence of a statistically significant association between any community‐level measure of stigma and HIV incidence (Figure 2).

## 4 DISCUSSION

In secondary analysis of data from a large cluster‐randomized trial in 21 communities in Zambia and South Africa, we found that a substantial number of HIV‐negative participants in the communities perceived stigma to be present in both the community and health settings, and, in some cases, held attitudes linked to fear and judgement of PLHIV. These individuals were not at greater risk of HIV infection compared to others in the community. In these same communities, a high proportion of PLHIV reported experiencing stigma (33.2% and 23.2% in Zambia and South Africa, respectively), while HWs, on average, “disagreed” with items on perception of stigma in communities and health settings. There was variation across communities, and differences between the two countries, in the level of reported stigma. However, we also found no evidence that the community‐level HIV incidence rate was associated with these community‐level measures of HIV stigma.

The literature on the association between HIV stigma and risk behaviour, access to prevention services and HIV incidence is much less developed [19] than that on PLHIV and access to diagnostic, care and treatment services. The HIV prevention cascade emphasizes three key components to support individuals from avoiding HIV acquisition: whether they are informed and motivated to adopt HIV prevention behaviours; whether they have readily accessible and available tools to them, such as condoms and pre‐exposure prophylaxis; and whether they have the capacity to enact the relevant behaviours [20]. At the individual level, one could argue that holding stigmatizing attitudes might limit motivation to enact prevention behaviours, while perceiving stigma in the community and health settings might limit motivation to access prevention tools or seek advice. A study in Cape Town bars found that participants agreeing with statements indicating AIDS‐related stigma reported higher levels of some risk behaviours [21]. In another study in Uganda, authors concluded that HIV risk was high among “boda boda” motorcyclists, was associated with HIV‐related stigma and that “interventions aimed at reducing HIV‐related stigma and alcohol use may potentially reduce the high rates of HIV transmission risk behavior” [22]. Data from Sierra Leone showed community‐level HIV disclosure concerns among women to be a driver of risky sex and self‐reported sexually transmitted infections [23]. Presumed HIV‐negative or unknown status individuals in China holding greater stigmatizing attitudes were more likely to be engaged in high‐risk behaviour [24, 25]. The study we present here was much larger than these previous studies, and measured HIV incident infection as the outcome. However, we did not have direct data to test these associations, but the fact that we see no overall impact of HIV stigma on HIV incidence might indicate that these prevention behaviours were less relevant in our context.

At the community level, if HIV stigma affects the steps of care in the treatment cascade, then this might have implications not only for PLHIV but also for those at risk of infection. In our previous work in this setting, we found limited evidence of an association between individual and community‐level stigma measures and the prevalence of viral suppression among PLHIV. The only exception was for those who reported higher internalized stigma and who were less likely to be virally suppressed [15]. In this paper, our community‐level analyses were intended to identify an association through the combined pathway of any effect of stigma on behaviour of those at risk of HIV infection as well as any impact on the likelihood that PLHIV may not be virally suppressed and/or having condomless sex. In the context of the literature, some may find the lack of any effect of HIV stigma on HIV incidence surprising. More large‐scale studies in other contexts would help deepen the research evidence base.

It is important to note that this analysis did not include items to measure the impacts of broader prejudice and discrimination experienced by a range of vulnerable and marginalized groups who in many settings may be at higher risk of HIV infection, including adolescent girls and young women, men who have sex with men, transgender people and female sex workers. We have published from our HW cohort on significant stigmatizing attitudes to some of these populations but did not include these items in this analysis because they were not asked of the participants [26]. These “key populations” experience overlapping, or intersectional, stigma and discrimination on the basis of their behaviour [27]. In some settings and for some populations, this also overlaps with socio‐economic inequalities along gender and race/ethnicity lines. HIV prevention services and health promotion efforts require targeted efforts and sustained support if they are to reach and be most impactful among these groups.

Our study was conducted among a large, representative random sample of community members in 21 communities who were followed‐up for up to 3 years to measure the risk of new HIV infection. We used theory‐based, harmonized and validated measures of a range of domains of stigma across three different populations.

Nevertheless, our study had limitations. First, despite many years of research into measurement of stigma, and our use of best‐practice measures, it remains a complex and evolving phenomenon, potentially subject to reporting biases. Therefore, the items we included to assess stigma may not have captured all the most important domains of stigma in our setting. Second, the communities we included were not randomly selected, or representative of the wide range of different types of community‐level stigma that may be experienced. While there was a large amount of variation between communities in some aspects of stigma, there was less for others, limiting our capacity to explore associations. Third, these are secondary analyses of data collected for another purpose, and uncontrolled confounding may mask some true associations. Lastly, other intersecting stigmas that we did not measure, such as sexual behaviour stigma or key population stigma, may influence HIV incidence more strongly than HIV stigma.

What would be the policy implications if further research in other contexts confirmed no association between HIV stigma and risk of new HIV infection in other sub‐Saharan African settings? This is good news in some ways—while stigma is a pernicious force that reduces the quality of life and health of PLHIV, its effects may not extend to heightening the risk of HIV infection. Efforts to eradicate HIV stigma are essential and must be redoubled for those already living with HIV and for those involved in HIV services, but these may not alone contribute to reducing the burden of new HIV infections. Societal enabling approaches to reduce HIV stigma and discrimination as well as remove legal barriers, reduce inequalities, improve gender equality and improve institutional and community structures will be needed to improve the effectiveness of HIV programmes and HIV outcomes [28]. Alternatively, further research in this area may help to identify which domains of stigma, under which conditions, do have a significant impact on HIV incidence, which would enable more optimized intervention design. For example, one area of growing importance is the emergence of reports of sigma related to a key HIV prevention tool, oral pre‐exposure prophylaxis [29, 30, 31, 32]. Community and clinic‐based discussions, adherence clubs and activities normalizing sexual behaviour and HIV prevention are all critical components of the response.

## 5 CONCLUSIONS

Our comprehensive analysis found no evidence of an association between HIV stigma and HIV incidence in the setting for the HPTN 071 “PopART” trial in Zambia and South Africa. Efforts to reduce new HIV infections and improve HIV prevention and treatment programmes considering HIV stigma in isolation may fail if not complemented by combination HIV prevention, with its biomedical, behavioural and structural components and person‐centred, community‐led approaches addressing all societal enablers of HIV, including stigma and discrimination. Continued scale up and strengthening of efforts to support the cascade of HIV prevention by increasing motivation to avoid HIV infection and use HIV prevention tools, removing barriers to access and empowering users to effectively use these tools over time are critical.

## COMPETING INTERESTS

There are no competing interests.

## AUTHORS’ CONTRIBUTIONS

JRH, GH, AS and VB conceptualized the manuscript. TP conducted the analysis with support from JRH and EW. TP, TM and CM‐M oversaw in‐country data collection of the health worker data set. NFB‐M, EW, RD, ASc and DD managed the PC data sets. TP, CM‐M and TM managed the health worker data sets. JH led the manuscript writing and conducted the literature review. RJH, SF, HA, PB and DD designed and led the cluster‐randomized trial and population cohort study within which the study is nested. JS provided guidance and oversight to social science research within the trial. EP‐M and YA oversaw the laboratory testing. GH and VB were responsible for the in‐country management, including data collection, and with JRH and AS designed the questions on stigma included in this analysis and are co‐investigators on the study protocol. All authors contributed to the writing of the article and have agreed the final draft for submission.

## DISCLAIMER

The content is solely the responsibility of the authors and does not necessarily represent the official views of the NIAID, NIMH, NIDA, PEPFAR, 3ie or the Bill & Melinda Gates Foundation.

## FUNDING

HPTN 071 (PopART) was sponsored by the National Institute of Allergy and Infectious Diseases (NIAID) under Cooperative Agreements UM1‐AI068619, UM1‐AI068617 and UM1‐AI068613, with funding from the US President's Emergency Plan for AIDS Relief (PEPFAR). Additional funding was provided by the International Initiative for Impact Evaluation (3ie) with support from the Bill & Melinda Gates Foundation, as well as by NIAID, the National Institute on Drug Abuse (NIDA) and the National Institute of Mental Health (NIMH), all part of NIH. The stigma ancillary study was funded by NIMH.

JRH, TP, JS and AS were members of the STRIVE consortium, which produced research on the structural drivers of HIV, including stigma. The STRIVE consortium was funded by UKaid from the Department for International Development (http://strive.lshtm.ac.uk/). However, the views expressed do not necessarily reflect the department's official policies. RJH and SF received funding from the UK Medical Research Council (MRC) and the UK Foreign, Commonwealth and Development Office (FCDO) under the MRC/FCDO Concordat agreement and is also part of the EDCTP2 programme supported by the European Union (MR/R010161/1). JRH, TP, GH, TM, VB and AS are funded by the Bill & Melinda Gates Foundation. This work was supported, in whole or in part, by the Bill & Melinda Gates Foundation [INV‐005239]. Under the grant conditions of the Foundation, a Creative Commons Attribution 4.0 Generic License has already been assigned to the Author Accepted Manuscript version that might arise from this submission.

## Supporting information


**Figure S1**. Study timelines for the HPTN 071 (PopART) cluster randomized trial and the stigma ancillary study.

**Figure S2**. Probability of seroconversion between PC0 and PC36 by stigma measures and study arm among 8172 participants.

**Figure S3**. Probability of seroconversion between PC0 and PC36 by stigma measures and age groups among 8172 participants.

**Table S1**. Description of stigma exposure variables.
**Table S2**. Summary characteristics of the two study samples, by country.
**Table S3**. Association between individual HIV stigma statements and HIV incidence (PC0–PC36) in the individual‐level analysis cohort (*n* = 8172).

## Data Availability

The data archive is held at Fred Hutch Cancer Center, Seattle, WA, USA. Requests can be sent to HPTN‐Data‐Access@scharp.org.

## References

[1] Ahmed S , Autrey J , Katz IT , Fox MP , Rosen S , Onoya D , et al. Why do people living with HIV not initiate treatment? A systematic review of qualitative evidence from low‐ and middle‐income countries. Soc Sci Med. 2018;213:72–84. 30059900 10.1016/j.socscimed.2018.05.048 PMC6813776

[2] Katz IT , Ryu AE , Onuegbu AG , Psaros C , Weiser SD , Bangsberg DR , et al. Impact of HIV‐related stigma on treatment adherence: systematic review and meta‐synthesis. J Int AIDS Soc. 2013;16(3 Suppl 2):18640. 24242258 10.7448/IAS.16.3.18640 PMC3833107

[3] Musheke M , Ntalasha H , Gari S , Mckenzie O , Bond V , Martin‐Hilber A , et al. A systematic review of qualitative findings on factors enabling and deterring uptake of HIV testing in sub‐Saharan Africa. BMC Public Health. 2013;13:220. 23497196 10.1186/1471-2458-13-220 PMC3610106

[4] Armstrong‐Mensah E , Hernandez P , Huka M , Suarez A , Akosile A , Joseph A , et al. HIV stigma among women and adolescent girls in South Africa: removing barriers to facilitate prevention. Madridge J AIDS. 2019;3(1):69–74.

[5] Turan JM , Nyblade L. HIV‐related stigma as a barrier to achievement of global PMTCT and maternal health goals: a review of the evidence. AIDS Behav. 2013;17(7):2528–39. 23474643 10.1007/s10461-013-0446-8

[6] Horter S , Bernays S , Thabede Z , Dlamini V , Kerschberger B , Pasipamire M , et al. “I don't want them to know”: how stigma creates dilemmas for engagement with treat‐all HIV care for people living with HIV in Eswatini. Afr J AIDS Res. 2019;18(1):27–37. 30782082 10.2989/16085906.2018.1552163

[7] Turan B , Hatcher AM , Weiser SD , Johnson MO , Rice WS , Turan JM . Framing mechanisms linking HIV‐related stigma, adherence to treatment, and health outcomes. Am J Public Health. 2017;107(6):863–9. 28426316 10.2105/AJPH.2017.303744 PMC5425866

[8] Bessong PO , Matume ND , Tebit DM . Potential challenges to sustained viral load suppression in the HIV treatment programme in South Africa: a narrative overview. AIDS Res Ther. 2021;18(1):1. 33407664 10.1186/s12981-020-00324-w PMC7788882

[9] Reynolds LJ , Camlin CS , Ware NC , Seeley J. Exploring critical questions for the implementation of “universal test and treat” approaches to HIV prevention and care. AIDS Care. 2016;28(Suppl 3):1–6. 10.1080/09540121.2016.1178960 27421046

[10] Arrington‐Sanders R , Hailey‐Fair K , Wirtz AL , Morgan A , Brooks D , Castillo M , et al. Role of structural marginalization, HIV stigma, and mistrust on HIV prevention and treatment among young Black Latinx men who have sex with men and transgender women: perspectives from youth service providers. AIDS Patient Care STDs. 2020;34(1):7–15. 31944853 10.1089/apc.2019.0165 PMC6983743

[11] Kim H‐Y , Grosso A , Ky‐Zerbo O , Lougue M , Stahlman S , Samadoulougou C , et al. Stigma as a barrier to health care utilization among female sex workers and men who have sex with men in Burkina Faso. Ann Epidemiol. 2018;28(1):13–19. 29425532 10.1016/j.annepidem.2017.11.009

[12] Stangl AL , Pliakas T , Mainga T , Steinhaus M , Mubekapi‐Musadaidzwa C , Viljoen L , et al. The effect of universal testing and treatment on HIV stigma in 21 communities in Zambia and South Africa. AIDS. 2020;34(14):2125–35. 32773484 10.1097/QAD.0000000000002658 PMC8425632

[13] Hayes R , Ayles H , Beyers N , Sabapathy K , Floyd S , Shanaube K , et al. HPTN 071 (PopART): rationale and design of a cluster‐randomised trial of the population impact of an HIV combination prevention intervention including universal testing and treatment — a study protocol for a cluster randomised trial. Trials. 2014;15:57. 24524229 10.1186/1745-6215-15-57 PMC3929317

[14] Hayes RJ , Donnell D , Floyd S , Mandla N , Bwalya J , Sabapathy K , et al. Effect of universal testing and treatment on HIV incidence ‐ HPTN 071 (PopART). N Engl J Med. 2019;381(3):207–18. 31314965 10.1056/NEJMoa1814556 PMC6587177

[15] Hargreaves JR , Pliakas T , Hoddinott G , Mainga T , Mubekapi‐Musadaidzwa C , Donnell D , et al. HIV stigma and viral suppression among people living with HIV in the context of universal test and treat: analysis of data from the HPTN 071 (PopART) trial in Zambia and South Africa. J Acquir Immune Defic Syndr. 2020;85(5):561–70. 32991336 10.1097/QAI.0000000000002504 PMC7654947

[16] Hargreaves JR , Stangl A , Bond V , Hoddinott G , Krishnaratne S , Mathema H , et al. HIV‐related stigma and universal testing and treatment for HIV prevention and care: design of an implementation science evaluation nested in the HPTN 071 (PopART) cluster‐randomized trial in Zambia and South Africa. Health Policy Plan. 2016;31(10):1342–54. 27375126 10.1093/heapol/czw071 PMC6702767

[17] Viljoen L , Mainga T , Casper R , Mubekapi‐Musadaidzwa C , Wademan DT , Bond VA , et al. Community‐based health workers implementing universal access to HIV testing and treatment: lessons from South Africa and Zambia‐HPTN 071 (PopART). Health Policy Plan. 2021;36(6):881–90. 33963387 10.1093/heapol/czab019 PMC8227454

[18] Stangl AL , Lilleston P , Mathema H , Pliakas T , Krishnaratne S , Sievwright K , et al. Development of parallel measures to assess HIV stigma and discrimination among people living with HIV, community members and health workers in the HPTN 071 (PopART) trial in Zambia and South Africa. J Int AIDS Soc. 2019;22(12):e25421. 31840400 10.1002/jia2.25421 PMC6912047

[19] Mahajan AP , Sayles JN , Patel VA , Remien RH , Sawires SR , Ortiz DJ , et al. Stigma in the HIV/AIDS epidemic: a review of the literature and recommendations for the way forward. AIDS. 2008;22(Suppl 2):S67–79. 10.1097/01.aids.0000327438.13291.62 PMC2835402 18641472

[20] Hargreaves JR , Delany‐Moretlwe S , Hallett TB , Johnson S , Kapiga S , Bhattacharjee P , et al. The HIV prevention cascade: integrating theories of epidemiological, behavioural, and social science into programme design and monitoring. Lancet HIV. 2016;3(7):e318–22. 27365206 10.1016/S2352-3018(16)30063-7

[21] Pitpitan EV , Kalichman SC , Eaton LA , Cain D , Sikkema KJ , Skinner D , et al. AIDS‐related stigma, HIV testing, and transmission risk among patrons of informal drinking places in Cape Town, South Africa. Ann Behav Med. 2012;43(3):362–71. 22367752 10.1007/s12160-012-9346-9 PMC3565536

[22] Nabifo SC , Tsai AC , Bajunirwe F. HIV‐related stigma and its association with HIV transmission risk behaviors among boda boda motorcyclists in Mbarara Municipality, southwestern Uganda. Int J STD AIDS. 2021;32(9):791–8. 33769905 10.1177/0956462420987760

[23] Daniel Kelly J , Reid MJ , Lahiff M , Tsai AC , Weiser SD. Community‐level HIV stigma as a driver for HIV transmission risk behaviors and sexually transmitted diseases in Sierra Leone: a population‐based study. J Acquir Immune Defic Syndr. 2017;75(4):399–407. 28406807 10.1097/QAI.0000000000001418 PMC5524569

[24] Chen J , Choe MK , Chen S , Zhang S . Community environment and HIV/AIDS‐related stigma in China. AIDS Educ Prev. 2005;17(1):1–11. 10.1521/aeap.17.1.1.58689 15843106

[25] Liu H , Hu Z , Li X , Stanton B , Naar‐King S , Yang H. Understanding interrelationships among HIV‐related stigma, concern about HIV infection, and intent to disclose HIV serostatus: a pretest‐posttest study in a rural area of eastern China. AIDS Patient Care STDs. 2006;20(2):133–42. 16475894 10.1089/apc.2006.20.133

[26] Krishnaratne S , Bond V , Stangl A , Pliakas T , Mathema H , Lilleston P , et al. Stigma and judgment toward people living with HIV and key population groups among three cadres of health workers in South Africa and Zambia: analysis of data from the HPTN 071 (PopART) trial. AIDS Patient Care STDs. 2020;34(1):38–50. 31944852 10.1089/apc.2019.0131 PMC6983735

[27] Turan JM , Elafros MA , Logie CH , Banik S , Turan B , Crockett KB , et al. Challenges and opportunities in examining and addressing intersectional stigma and health. BMC Med. 2019;17(1):7. 30764816 10.1186/s12916-018-1246-9 PMC6376691

[28] Stangl AL , Pliakas T , Izazola‐Licea JA , Ayala G , Beattie TS , Ferguson L , et al. Removing the societal and legal impediments to the HIV response: an evidence‐based framework for 2025 and beyond. PLoS One. 2022;17(2):e0264249. 35192663 10.1371/journal.pone.0264249 PMC8863250

[29] Biello KB , Oldenburg CE , Mitty JA , Closson EF , Mayer KH , Safren SA , et al. The “safe sex” conundrum: anticipated stigma from sexual partners as a barrier to PrEP use among substance using MSM engaging in transactional sex. AIDS Behav. 2017;21(1):300–6. 27351194 10.1007/s10461-016-1466-y PMC5624223

[30] Calabrese SK , Dovidio JF , Tekeste M , Taggart T , Galvao RW , Safon CB , et al. HIV pre‐exposure prophylaxis stigma as a multidimensional barrier to uptake among women who attend planned parenthood. J Acquir Immune Defic Syndr. 2018;79(1):46–53. 29847480 10.1097/QAI.0000000000001762 PMC6092222

[31] Reisner SL , Moore CS , Asquith A , Pardee DJ , Sarvet A , Mayer G , et al. High risk and low uptake of pre‐exposure prophylaxis to prevent HIV acquisition in a national online sample of transgender men who have sex with men in the United States. J Int AIDS Soc. 2019;22(9):e25391. 31536171 10.1002/jia2.25391 PMC6752156

[32] Velloza J , Khoza N , Scorgie F , Chitukuta M , Mutero P , Mutiti K , et al. The influence of HIV‐related stigma on PrEP disclosure and adherence among adolescent girls and young women in HPTN 082: a qualitative study. J Int AIDS Soc. 2020;23(3):e25463. 32144874 10.1002/jia2.25463 PMC7060297

