# Supplementary figures and images for: The association between HIV stigma and HIV incidence in the context of universal testing and treatment: analysis of data from the HPTN 071 (PopART) trial in Zambia and South Africa
Source: J Int AIDS Soc. 2022 Jul 12;25(Suppl 1):e25931. doi: 10.1002/jia2.25931 (PMC9274206; doi:10.1002/jia2.25931)

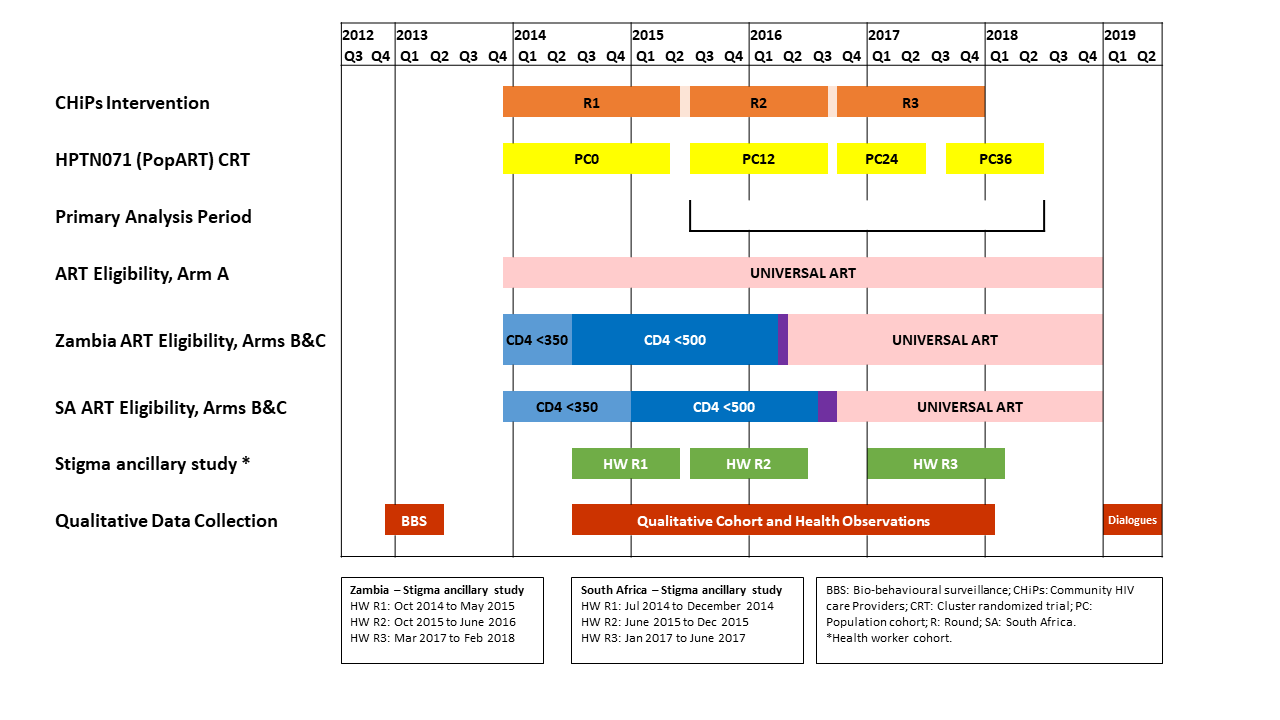

Supplement: Supplementary file 1 — Figure S1. Study timelines for the HPTN 071 (PopART) cluster randomized trial and the stigma ancillary study. [file JIA2-25-e25931-s002.png]

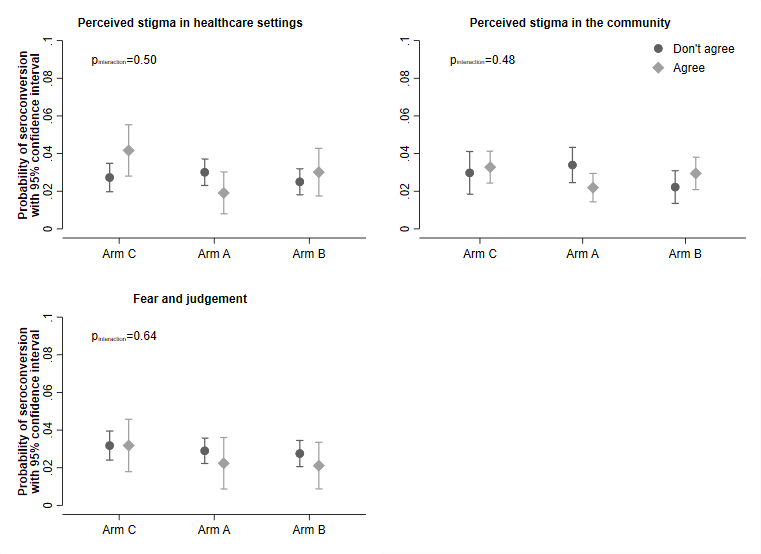

Supplement: Supplementary file 2 — Figure S2. Probability of seroconversion between PC0 and PC36 by stigma measures and study arm among 8172 participants. [file JIA2-25-e25931-s003.png]

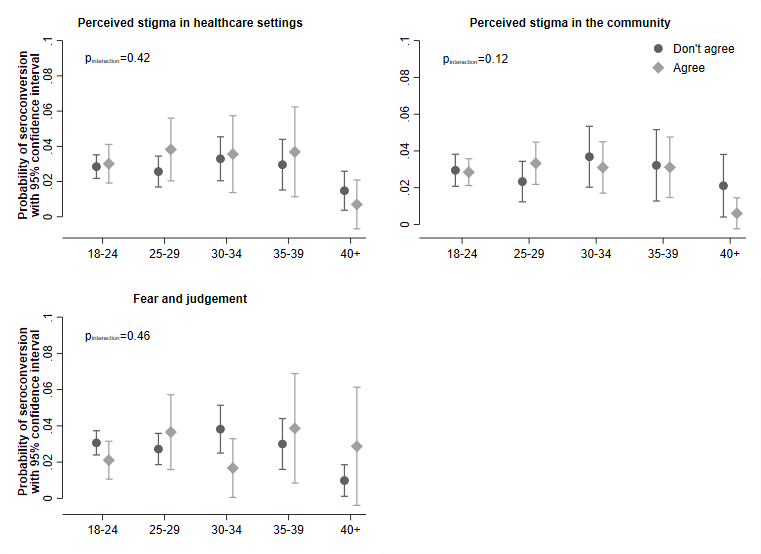

Supplement: Supplementary file 3 — Figure S3. Probability of seroconversion between PC0 and PC36 by stigma measures and age groups among 8172 participants. [file JIA2-25-e25931-s004.png]
