# Supplementary material for: The association between HIV stigma and HIV incidence in the context of universal testing and treatment: analysis of data from the HPTN 071 (PopART) trial in Zambia and South Africa
Source: J Int AIDS Soc. 2022 Jul 12;25(Suppl 1):e25931. doi: 10.1002/jia2.25931 (PMC9274206; doi:10.1002/jia2.25931)
Supplement: Supplementary file 4 — Table S1. Description of stigma exposure variables. Table S2. Summary characteristics of the two study samples, by country. Table S3. Association between individual HIV stigma statements and HIV incidence (PC0–PC36) in the individual‐level analysis cohort (n = 8172). [file JIA2-25-e25931-s001.docx]

**Supplementary files**

**Table S1.** Description of stigma exposure variables.

| **Population group and exposure** | **Stigma items / statements** |
| --- | --- |
| **(People living with HIV)** |  |
| *Any reported internalized stigma (Three items)* | (1) I have lost respect or standing in the community because of my HIV status  (2) I think less of myself because of my HIV status  (3) I have felt ashamed because of my HIV status |
| *Any reported experienced stigma in the community (Five items)* | (1) People have talked badly about me because of my HIV status  (2) Someone else disclosed my HIV status without my permission  (3) I have been verbally insulted, harassed and/or threatened because of my HIV status  (4) I have been physically assaulted because of my HIV status  (5) I have felt that people have not wanted to sit next to me, for example, on public transport, at church or in a waiting room because of my HIV status |
| *Any reported experienced stigma in health service settings (Three items)* | (1) I have been denied health services because of my HIV status  (2) Healthcare workers talked badly about me because of my HIV status  (3) A health worker disclosed my HIV status without my permission |
| *Any stigma (Eleven items)* | All items above |
| **(Community members not living with HIV)** | |
| *Any negative attitudes (Fear and judgment using three items)* | (1) I fear that I could contract HIV if I come into contact with the saliva of a person living with HIV  (2) I would not like to sit close to someone living with HIV, for example on public transport, at church or in a waiting room  (3) I would be ashamed if someone in my family had HIV |
| *Any perceived stigma in community settings (Five items)* | (1) People thought to be living with HIV are sometimes physically assaulted  (2) People sometimes talk badly about PLHIV to others  (3) People thought to be living with HIV lose respect or standing  (4) People thought to be living with HIV are verbally insulted, harassed and/or threatened  (5) People sometimes disclose that other people are HIV positive without their permission |
| *Any perceived stigma in health service settings (Two items)* | (1) Health workers sometimes talk badly about people living with or thought to be living with HIV to others  (2) Health workers sometimes disclose that other people are HIV positive without their permission |
| **Health Workers self-reporting not living with HIV** | |
| *Any negative attitudes (Fear and judgment using five items)* | (1) I fear that I could contract HIV if I come into contact with the saliva of a person living with HIV  (2) I avoid physical contact with clients living with HIV  (3) HIV is punishment from God  (4) Other people deserve access to health services more than PLHIV  (5) I would be ashamed if someone in my family had HIV |
| *Any perceived stigma in the community (Five items)* | (1) People thought to be living with HIV are sometimes physically assaulted  (2) People sometimes talk badly about PLHIV to others  (3) People thought to be living with HIV lose respect or standing  (4) People thought to be living with HIV are verbally insulted, harassed, or threatened  (5) People hesitate to start ARV drugs because they are afraid others will learn they are living with HIV |
| *Any perceived co-worker stigma (Four items)* | (1) My co-workers sometimes talk badly about people thought to be living with HIV  (2) My co-workers sometimes gossip about clients' HIV test results  (3) My co-workers sometimes treat people living with HIV poorly when providing them with health services  (4) My co-workers sometimes verbally insult clients living with HIV |

**Table S2**. Summary characteristics of the two study samples, by country.

|  | **Cohort-level analysis cohort** | | | | | |  | **Individual-level analysis cohort** | | | | | |
| --- | --- | --- | --- | --- | --- | --- | --- | --- | --- | --- | --- | --- | --- |
|  | **Zambia**  **(n=15,238)** | | **South Africa**  **(n=10,872)** | | **Total**  **(n=26,110)** | |  | **Zambia**  **(n=4766)** | | **South Africa**  **(n=3406)** | | **Total**  **(n=8172)** | |
|  | **No.** | **%** | **No.** | **%** | **No.** | **%** |  | **No.** | **%** | **No.** | **%** | **No.** | **%** |
| **Sex** |  |  |  |  |  |  |  |  |  |  |  |  |  |
| Male | 4400 | 28.88 | 3657 | 33.64 | 8057 | 30.86 |  | 1335 | 28.01 | 1064 | 31.24 | 2399 | 29.36 |
| Female | 10838 | 71.12 | 7215 | 66.36 | 18053 | 69.14 |  | 3431 | 71.99 | 2342 | 68.76 | 5773 | 70.64 |
| **Age group (at PC0)** |  |  |  |  |  |  |  |  |  |  |  |  |  |
| 16-24 | 7948 | 52.16 | 4191 | 38.55 | 12139 | 46.49 |  | 2364 | 49.60 | 1271 | 37.32 | 3635 | 44.48 |
| 25-29 | 3027 | 19.86 | 2305 | 21.20 | 5332 | 20.42 |  | 993 | 20.84 | 752 | 22.08 | 1745 | 21.35 |
| 30-34 | 2029 | 13.32 | 1816 | 16.70 | 3845 | 14.73 |  | 665 | 13.95 | 555 | 16.29 | 1220 | 14.93 |
| 35-39 | 1389 | 9.12 | 1347 | 12.39 | 2736 | 10.48 |  | 447 | 9.38 | 428 | 12.57 | 875 | 10.71 |
| 40+ | 845 | 5.55 | 1213 | 11.16 | 2058 | 7.88 |  | 297 | 6.23 | 400 | 11.74 | 697 | 8.53 |
| **Education (reported at first visit)** |  |  |  |  |  |  |  |  |  |  |  |  |  |
| Did not complete secondary | 4400 | 28.88 | 1367 | 12.57 | 5767 | 22.09 |  | 1400 | 29.37 | 426 | 12.51 | 1826 | 22.34 |
| Completed secondary | 9663 | 63.41 | 8944 | 82.27 | 18607 | 71.26 |  | 3009 | 63.13 | 2813 | 82.59 | 5822 | 71.24 |
| Further | 1175 | 7.71 | 561 | 5.16 | 1736 | 6.65 |  | 357 | 7.49 | 167 | 4.90 | 524 | 6.41 |
| **Marital status (at enrolment)** |  |  |  |  |  |  |  |  |  |  |  |  |  |
| Married or living as married | 7963 | 52.26 | 3019 | 27.77 | 10982 | 42.06 |  | 2532 | 53.13 | 971 | 28.51 | 3503 | 42.87 |
| Never married | 6001 | 39.38 | 7599 | 69.90 | 13600 | 52.09 |  | 1832 | 38.44 | 2361 | 69.32 | 4193 | 51.31 |
| Divorced, separated, or widowed | 1274 | 8.36 | 254 | 2.34 | 1528 | 5.85 |  | 402 | 8.43 | 74 | 2.17 | 476 | 5.82 |

**Table S3**. Association between individual HIV stigma statements and HIV Incidence (PC0-PC36) in the individual-level analysis cohort (n=8,172).

| **Stigma measures** | **Unadjusted IRR** | **Adjusted IRR*** | **Adjusted IRR**** |
| --- | --- | --- | --- |
| **Perceived stigma in the community** |  |  |  |
| Score | 0.88 (0.71-1.10) | 0.89 (0.71-1.11) | 0.89 (0.72-1.11) |
| Individual stigma statements |  |  |  |
| *People sometimes talk badly about people living with or thought to be living with HIV to others* |  |  |  |
| Don't agree | 1 | 1 | 1 |
| Agree | 0.93 (0.71-1.23) | 0.94 (0.71-1.24) | 0.95 (0.72-1.25) |
| *People living with or thought to be living with HIV lose respect or standing* |  |  |  |
| Don't agree | 1 | 1 | 1 |
| Agree | 0.97 (0.72-1.31) | 0.98 (0.73-1.33) | 0.99 (0.73-1.33) |
| *People living with or thought to be living with HIV are verbally insulted, harassed and/or threatened* |  |  |  |
| Don't agree | 1 | 1 | 1 |
| Agree | **0.71 (0.50-0.99)** | 0.71 (0.51-1.00) | 0.72 (0.51-1.01) |
| *People living with or thought to be living with HIV are sometimes physically assaulted* |  |  |  |
| Don't agree | 1 | 1 | 1 |
| Agree | 0.94 (0.66-1.34) | 0.94 (0.66-1.34) | 0.94 (0.66-1.34) |
| *People sometimes disclose that other people are HIV positive without their permission* |  |  |  |
| Don't agree | 1 | 1 | 1 |
| Agree | 0.77 (0.58-1.02) | 0.78 (0.58-1.04) | 0.78 (0.58-1.04) |
| **Perceived stigma in healthcare settings** |  |  |  |
| Score | 0.97 (0.79-1.19) | 0.98 (0.80-1.21) | 0.98 (0.80-1.20) |
| Individual stigma statements |  |  |  |
| *Health workers sometimes talk badly about people living with or thought to be living with HIV to others* |  |  |  |
| Don't agree | 1 | 1 | 1 |
| Agree | 1.16 (0.85-1.59) | 1.19 (0.87-1.64) | 1.18 (0.86-1.62) |
| *Health workers sometimes disclose that other people are HIV positive without their permission* |  |  |  |
| Don't agree | 1 | 1 | 1 |
| Agree | 1.05 (0.75-1.48) | 1.06 (0.76-1.49) | 1.06 (0.76-1.49) |
| **Fear and judgement** |  |  |  |
| Score | 0.92 (0.73-1.17) | 0.92 (0.73-1.17) | 0.92 (0.73-1.17) |
| Individual stigma statements |  |  |  |
| *I would be ashamed if someone in my family had HIV* |  |  |  |
| Don't agree | 1 | 1 | 1 |
| Agree | 0.95 (0.61-1.47) | 0.96 (0.62-1.49) | 0.98 (0.63-1.52) |
| *I would not like to sit close to someone living with HIV, for example on public transport, at church or in a waiting room* |  |  |  |
| Don't agree | 1 | 1 | 1 |
| Agree | 0.81 (0.49-1.34) | 0.81 (0.49-1.34) | 0.79 (0.48-1.32) |
| *I fear that I could contract HIV if I come into contact with the saliva of a person living with HIV* |  |  |  |
| Don't agree | 1 | 1 | 1 |
| Agree | 0.82 (0.58-1.17) | 0.83 (0.59-1.17) | 0.83 (0.59-1.17) |

IRR: Incidence Rate Ratio; PC: Population cohort.

*Adjusted for sex and age group; ** Adjusted for sex, age group, marital status and education.

All models were developed within a Poisson regression framework adjusted using community as a fixed term.

P<0.05 highlighted in bold.


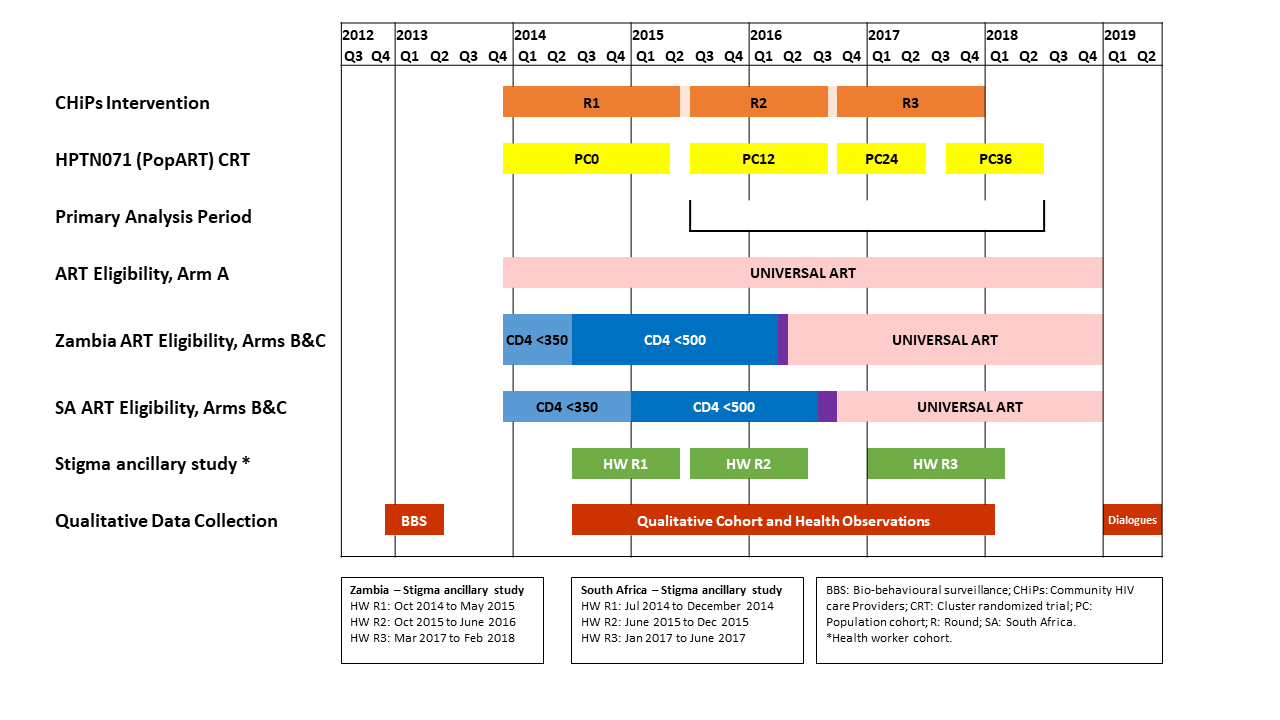


**Figure S1.** Study timelines for the HPTN 071 (PopART) cluster randomizes trial and the stigma ancillary study.

**
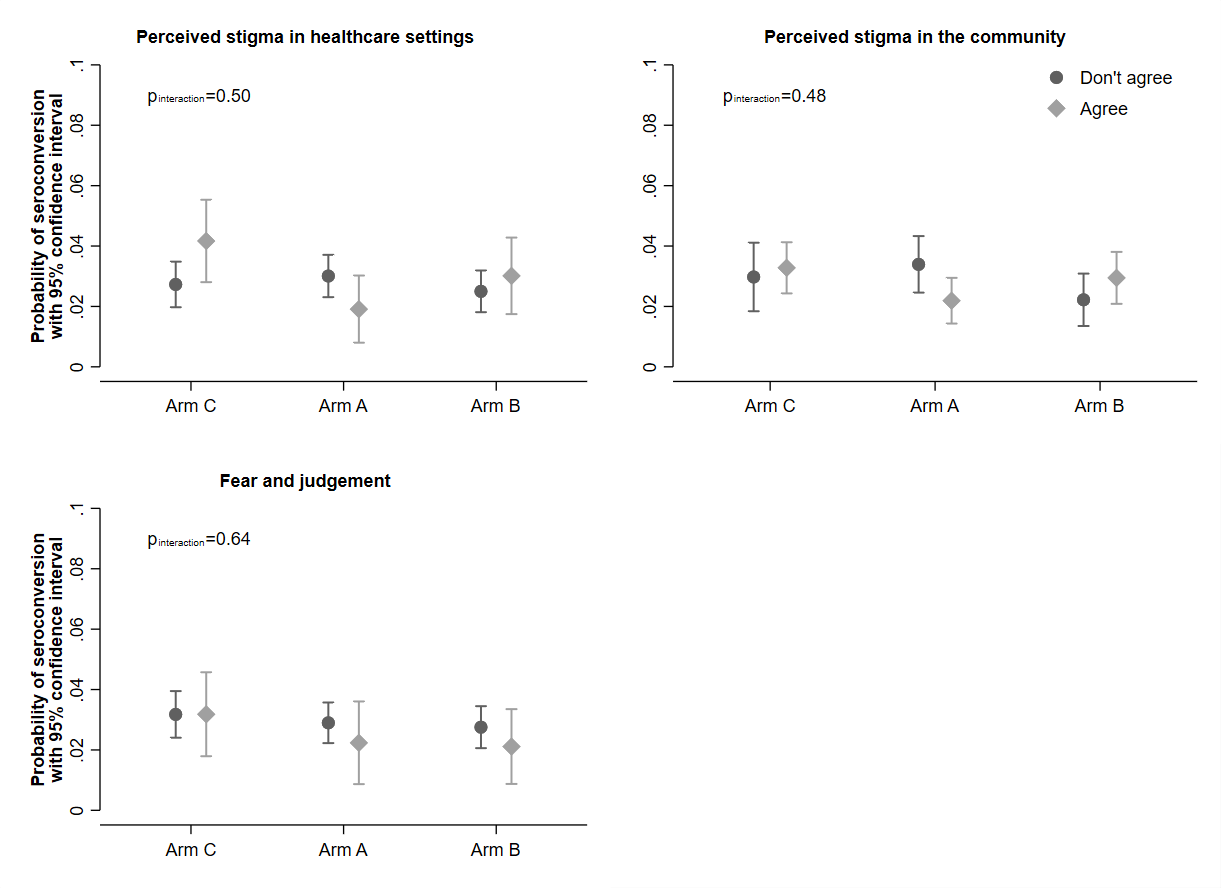
Figure S2.** Probability of seroconversion between PC0 and PC36 by stigma measures and study arm among 8,172 participants.

**
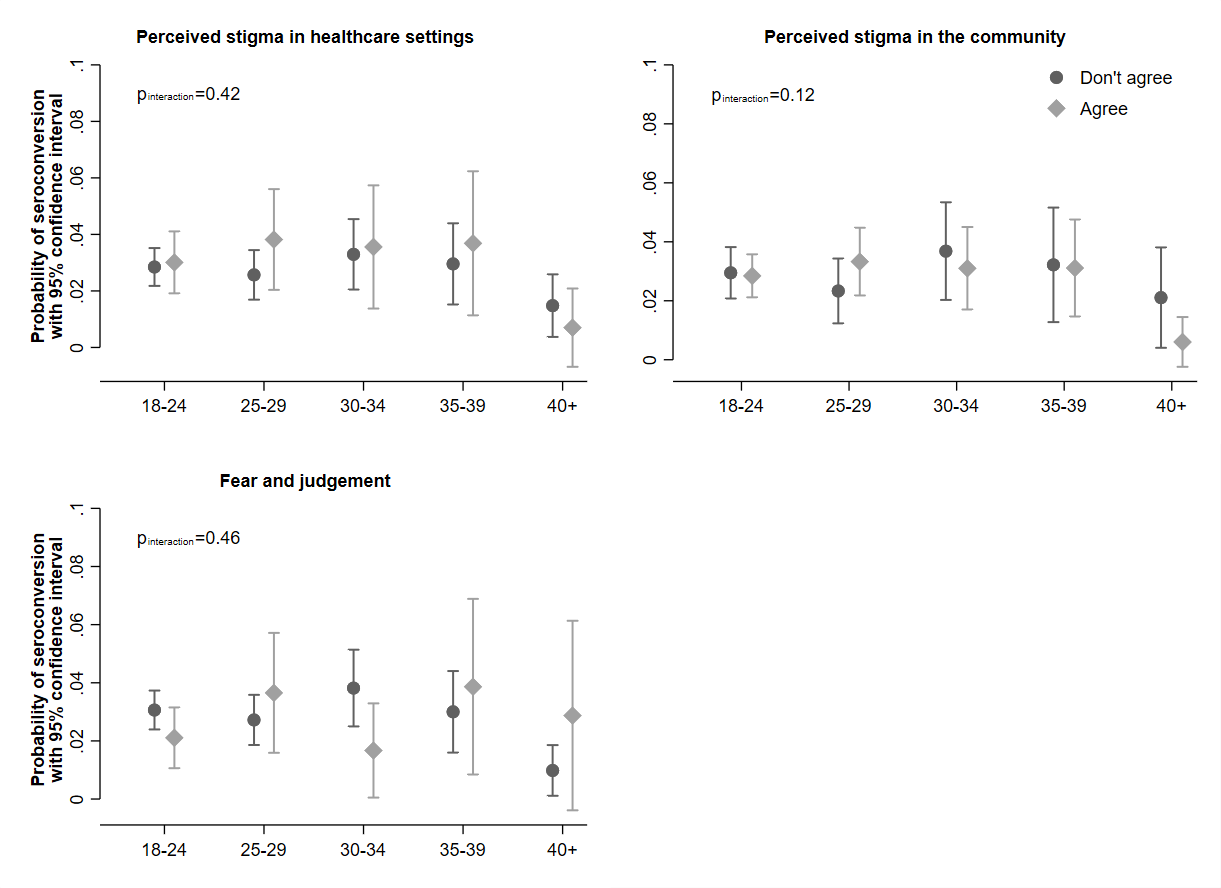
Figure S3.** Probability of seroconversion between PC0 and PC36 by stigma measures and age groups among 8,172 participants.
